# The Atlanta Mosquito Crusade

**Published:** 1903-07

**Authors:** 


### THE ATLANTA MOSQUITO CRUSADE.

DR. J. P. KENNEDY, the Health Officer, with the assistance of Dr. H. F. Harris, Professor of Pathology and Bacteriology at the Atlanta College of Physicians and Surgeons, has inaugurated a war against mosquitoes.

Many kinds of mosquitoes are found here, the common culex being the most frequent. The anophiles or malaria-bearing mosquito is occasionally seen at different points in the city and its environs, and also the C. fasciatus or yellow fever mosquito. The former is roughly identified by the position assumed while at rest, an angle of 90° or greater to its support, and the later by the transverse crural stripes. Mosquitoes find their principal breeding-places in the residual water of sewer eyes, and, if not dislodged by flushing or destroyed, multiply in great numbers. Scattered refuse capable of containing water, as old cans, pans and pots, offers excellent media for breeding mosquitoes, though they do not breed when the water is freely exposed to sunlight. Kerosene oil, forming a floating pellicle, seems the most efficient agent for the destruction of breeding mosquitoes. It is introduced into the sewer traps and in places where stagnant water exists. The people of the city should second the efforts of the health authorities and observe backyard sanitation as well.

It is hoped that the coming summer will be made notable by the absence of that vocalization which has given to this dipterous insect the highly descriptive title of culex damnatus.

